# 1933. Characteristics of Community-Associated Extended-Spectrum β-Lactamase Producing *Enterobacterales* Infections in Monroe County, NY, 2020-2021

**DOI:** 10.1093/ofid/ofad500.093

**Published:** 2023-11-27

**Authors:** Julia Tellerman, Savanah Russ, Hsioa Che Looi, Rebecca Tsay, christopher J Myers, Ghinwa Dumyati

**Affiliations:** New York Emerging Infections Program, Rochester, NY; Center for Community Health & Prevention, University of Rochester Medical Center, Rochester, New York; New York Rochester Emerging Infections Program at the University of Rochester Medical Center, Rochester, New York, Rochester, New York; New York Rochester Emerging Infections Program at the University of Rochester Medical Center, Rochester, NY; University of Rochester, Rochester, New York; New York Emerging Infections Program and University of Rochester Medical Center, Rochester, New York

## Abstract

**Background:**

Extended-spectrum β-lactamase–producing *Enterobacterales* (ESBL) have emerged as a serious multidrug resistant threat to the community. Epidemiologic data for community-associated (CA) ESBL infections are limited compared to that for healthcare-associated (HA) infections. We examined characteristics of CA ESBL cases over a two-year period and compared them to those of HA cases to guide prevention.

**Methods:**

CA ESBL cases from 1/2020-12/2021 were identified via laboratory and population-based surveillance in Monroe County as part of the CDC Emerging Infections Program. Cases were defined as the first instance of positive culture in a county resident from a normally sterile site or urine of *Escherichia coli, Klebsiella pneumoniae*, or *K. oxytoca* resistant to ≥ 1 3rd-generation cephalosporin and nonresistant to carbapenems. Epidemiologic data were collected through medical record abstraction. Cases were classified as HA if they had prior healthcare exposures (e.g. hospitalization, surgery, chronic dialysis, indwelling devices, or external catheters) and CA if they did not. Case addresses were geocoded to the census tract level to obtain Social Vulnerability Index (SVI) percentiles. Chi-square analyses were conducted to quantify the difference in distribution of these characteristics between CA and HA cases.

**Results:**

From 2020-2021, 1729 incident cases were identified; 854 (49%) were classified as CA. Compared to HA cases, CA cases were younger (median 59 vs. 70 years), more likely to be female (89.8% vs 65.3%), less likely to have a urinary tract abnormality (27.0% vs 56.8%) or underlying comorbidities (73.2 % vs 95.8%), and less likely to have received prior antibiotics (24.0% vs 55.1%). Interestingly, CA cases were more widely spread across SVI quartiles compared to HA cases; a higher proportion of HA cases resided in census tracts in the 3rd or 4th SVI quartiles (Table 1).

**Figure:**
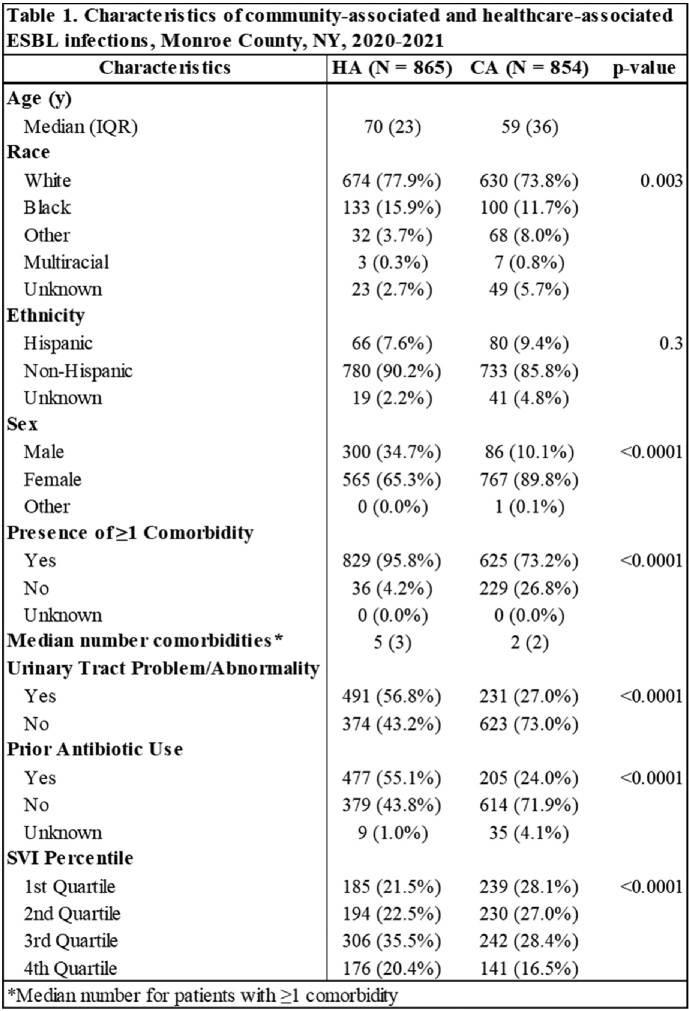

**Conclusion:**

Patient-specific risk factors classically associated with HA ESBL are less commonly associated with CA ESBL in Monroe County. Community-based preventions may need to target different populations than hospital-based strategies. As ESBL incidence increases, continued surveillance to identify at-risk groups and explore the association of specific social determinants of health is necessary.

**Disclosures:**

**Rebecca Tsay, MPH**, CDC: Grant/Research Support **Ghinwa Dumyati, MD**, Pfizer: Grant/Research Support

